# 7-Difluoromethoxyl-5,4′-di-n-octyl genistein inhibits the stem-like characteristics of gastric cancer stem-like cells and reverses the phenotype of epithelial-mesenchymal transition in gastric cancer cells

**DOI:** 10.3892/or.2022.8391

**Published:** 2022-08-22

**Authors:** Xiaozheng Cao, Kaiqun Ren, Zhengwei Song, Duo Li, Meifang Quan, Yu Zheng, Jianguo Cao, Wenbin Zeng, Hui Zou

Oncol Rep 36: 1157–1165, 2016; DOI: 10.3892/or.2016.4848

Subsequently to the publication of the above article, an interested reader drew to the authors' attention that there appeared to be two pairs of images in Fig. 2A and B on p. 1159 and Fig. 4 on p. 1161 that contained overlapping sections, such that these figures, which were intending to show the results from differently performed experiments, may have been derived from the same original sources.

The authors have examined their original data, and realize that, although Fig. 2 was correct as presented in the article, these data were erroneously and inadvertently included in Fig. 4. The revised version of Fig. 4, which shows the inhibition of sphere-forming ability by 7-difluoromethoxyl-5,4′-di-n-octyl genistein (DOFG) in gastric cancer stem-like cells derived from SGC-7901 cells, is shown below, now including the correct data for the panels showing treatment with 0 and 1.0 µmol/l DOFG, and with re-quantification of these data. The authors are grateful to the Editor of *Oncology Reports* for allowing them the opportunity to publish a Corrigendum, and all the authors agree with its publication. Furthermore, they apologize to the readership for any inconvenience caused.

## Figures and Tables

**Figure 4.**
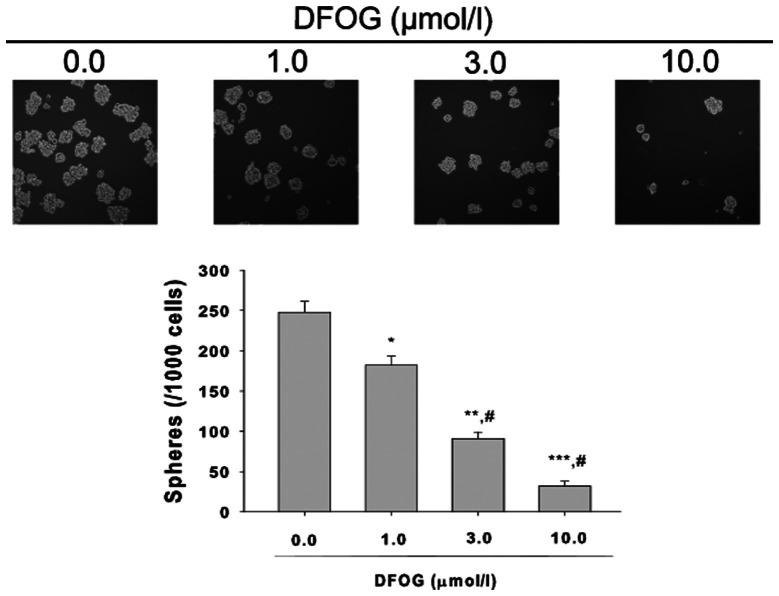
Inhibition of sphere-forming ability by DFOG in gastric cancer stem-like cells (GCSLCs) from SGC-7901 cells and the statistical analysis. *P<0.05 vs. the group treated with 0.1% DMSO; **P<0.01 vs. the group treated with 0.1% DMSO; ***P<0.001 vs. the group treated with 0.1% DMSO; #P<0.05 vs. the group treated with 1.0 µmol/l DFOG.

